# Nitrogen-Vacancy Color Centers Created by Proton Implantation in a Diamond

**DOI:** 10.3390/ma14040833

**Published:** 2021-02-09

**Authors:** Mariusz Mrózek, Mateusz Schabikowski, Marzena Mitura-Nowak, Janusz Lekki, Marta Marszałek, Adam M. Wojciechowski, Wojciech Gawlik

**Affiliations:** 1 Institute of Physics, Jagiellonian University, Łojasiewicza 11, 30-348 Kraków, Poland; a.wojciechowski@uj.edu.pl (A.M.W.); wojciech.gawlik@uj.edu.pl (W.G.); 2 Institute of Nuclear Physics, Polish Academy of Sciences, Radzikowskiego 152, 31-342 Kraków, Poland; mateusz.schabikowski@ifj.edu.pl (M.S.); marzena.mitura-nowak@ifj.edu.pl (M.M.-N.); Janusz.Lekki@ifj.edu.pl (J.L.); Marta.Marszalek@ifj.edu.pl (M.M.)

**Keywords:** diamond, proton implantation, nitrogen-vacancy

## Abstract

We present an experimental study of the longitudinal and transverse relaxation of ensembles of negatively charged nitrogen-vacancy (NV${}^{-}$) centers in a diamond monocrystal prepared by 1.8 MeV proton implantation. The focused proton beam was used to introduce vacancies at a 20 µm depth layer. Applied doses were in the range of $1.5\times 10^{13}$ to $1.5\times 10^{17}$ ions/cm${}^{2}$. The samples were subsequently annealed in vacuum which resulted in a migration of vacancies and their association with the nitrogen present in the diamond matrix. The proton implantation technique proved versatile to control production of nitrogen-vacancy color centers in thin films.

## 1. Introduction

Negatively charged nitrogen-vacancy (NV${}^{-}$) color centers in a diamond have been a focus of recent research due to their outstanding properties which make them promising candidates for fluorescent markers in biological systems [1,2] and for numerous applications e.g., in quantum information [3,4], magnetometry [5,6], and temperature sensing [7,8]. Successful application thus requires a sound knowledge of interaction of the NV${}^{-}$ centers with the environment. This motivates recent efforts to study the relaxation dynamics of the NV${}^{-}$ electron-spin polarization. Particularly, measurements of the longitudinal relaxation time $T_{1}$ (the decay lifetime for NVs population initialized to a ground-state magnetic sublevel) enable development of new techniques which use NV${}^{-}$ as a spin probe [9]. The spin phase coherence time ($T_{2}$) is a critical figure of merit for the emerging quantum-based applications—for example, for ensemble magnetometry, the sensitivity scales as ${\left(N_{S}T_{2}\right)}^{-1/2}$, where $N_{S}$ is the number of interrogated spins [10]. In quantum computing, $T_{2}$ constrains the minimum gate operation time and limits the performance of quantum error correction protocols [11]. The phase coherence time ($T_{2}$) is generally limited by stochastic processes, such as phonon interactions, which cause irreversible changes in the axial spin projection. Such processes are known as longitudinal relaxation with a characteristic timescale, $T_{1}$ [12].

For many sensing applications, thin and dense layers of NV centers are desired in order to maximize the signal while maintaining sufficiently short distance between the source and the sensing spins. Such layers have already been used for wide-field magnetic field imaging [13,14,15,16,17,18,19]. Although some NV centers and nitrogen atoms are always present in diamonds, the NV concentration can be substantially increased in a desired region by means of nitrogen ions’ implantation. A common alternative technique is the irradiation of the a priori nitrogen-rich sample (entire diamond or a doped layer) with electrons, protons, or ions [20,21,22,23]. It creates vacancies in the lattice and is followed by high-temperature (600 ${}^{\circ }$C) annealing that allows their migration and recombination with nitrogen present in the diamond sample. Importantly, the spatial- and ion-energy control of the implantation process enables tailoring of the local NV concentration distribution towards specific applications. As the implantation and irradiation processes cause lattice damage, particularly at high fluence, these processes should be well controlled to efficiently create NV centers while avoiding crystal damage and related spin-lifetime shortening. Continuous in-situ annealing during the irradiation/implantation has been used in Ref. [24] to enable efficient nitrogen to NV-center conversion with reduced vacancy clustering.

In this work, we analyze creation of NV centers using high-energy (1.8 MeV) proton implantation in nitrogen-rich ([N]∼50 ppm) diamonds. At such energies, protons penetrate up to ∼20 µm into the diamond, which is sufficient to create vacancies in typically used NV sensing layers that have a thickness of 0.1–10 µm. On the other hand, the limited proton range, as opposed to that of high-energy electrons (∼500µm at the energy of 5 MeV), efficiently reduces creation of vacancies and NV centers in the deeper layers of a bulk diamond, which may be favorable for limiting the background fluorescence. The work presented here builds upon the research carried out in Ref. [25]. A similar type of implantation was performed in [26] and the photoluminescence was studied. Here, we report on an investigation of the longitudinal $T_{1}$ and transverse $T_{2}$ spin relaxation times of NV ensembles as a function of proton implantation dose. This information is vital for numerous applications, for example in magnetometry [27].

## 2. Sample Preparation

The proton implantation was performed on two 3.0×3.0×0.3 mm${}^{3}$ sized type Ib mono-crystalline diamond samples synthesized by the high-pressure high-temperature (HPHT) technique. The (100)-oriented one side polished diamond samples were purchased from Element Six. The initial nitrogen concentration in both samples was $[N_{i}]∼50$ ppm.

Protons were implanted on the polished side of the samples using the ion beam from a Van de Graaff accelerator (located in the Institute of Nuclear Physics, Polish Academy of Sciences, Kraków, Poland) at the energy of 1.8 MeV with the spot of an approximately 20 µm diameter. The samples were labeled as HEN1 and HEN2. Detailed information about the preparation of sample HEN1 can be found in Ref. [25]. The doses applied to both samples are listed in Table 1. Spots 1–6 refer to the sample HEN1 and spots 7–8 to sample HEN2.

After implantation, the samples were annealed for 2 h in a vacuum system at about 900 ${}^{\circ }$C in order to increase the diffusion of vacancies and stimulate the formation of NV centers.

**Figure 1:**
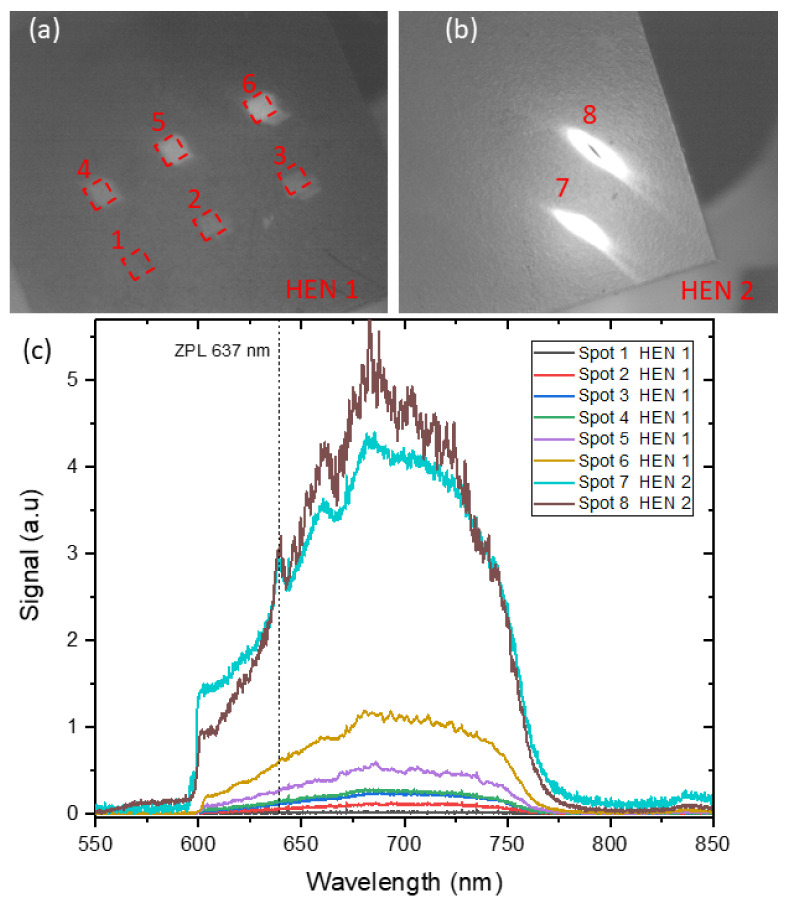
The proton-irradiated samples: (**a**) the sample HEN1 with six areas of the implanted doses in the range of $1.5\times 10^{13}$ to $1.5\times 10^{16}$ ions/cm${}^{2}$; (**b**) the sample HEN2 with two areas of the implanted dose range of $4.5\times 10^{16}$ to $1.5\times 10^{17}$ ions/cm${}^{2}$; (**c**) the fluorescence spectra from the marked individual spots. The zero-phonon line (ZPL) is marked with a dotted line.

Figure 1 shows photographs of two samples. Spots 1–6 and 7–8 are visible in Figure 1a,b, respectively. Spots 7–8 are characterized by the highest dose of protons. Figure 1c shows the fluorescence spectrum from individual marked points.

**Figure 2:**
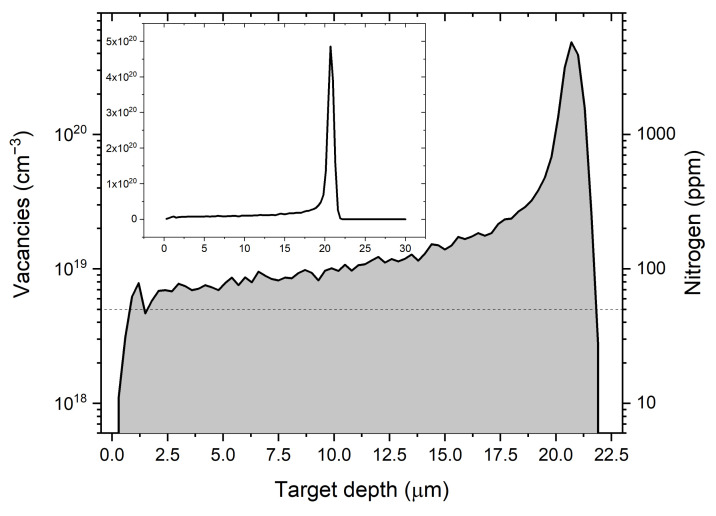
The graph shows the number of vacancies produced by proton irradiation with a dose $1.5\times 10^{16}$ cm${}^{-2}$ depending on the depth in the sample. It can be seen that the largest number of vacancies is generated at a depth of 20 µm. The dashed line indicates the amount of nitrogen in the tested samples. The inset shows the same plot but with a linear scale.

For the simulations of proton implantation in the diamond matrix, we used the SRIM 2013 suite [28] with the following parameters: ion energy 1.8 MeV, dose $1.5\times 10^{16}$ cm${}^{-2}$, atomic density 3.52 g/cm${}^{3}$, and the displacement threshold energy of 37.5 eV for the (100) direction (Ref. [29]). Figure 2 presents the simulated damage (created vacancies) due to the proton impact on the diamond sample for the dose of $1.5\times 10^{16}$ cm${}^{-2}$. As can be seen, for a 1.8 MeV proton beam, most vacancies are generated around the depth of 20 µm. The dashed line in Figure 2 shows the nitrogen content in the tested diamond samples. For this dose of protons, the concentration of created vacancies exceeds the nitrogen concentration everywhere up to this depth.

**Figure 3:**
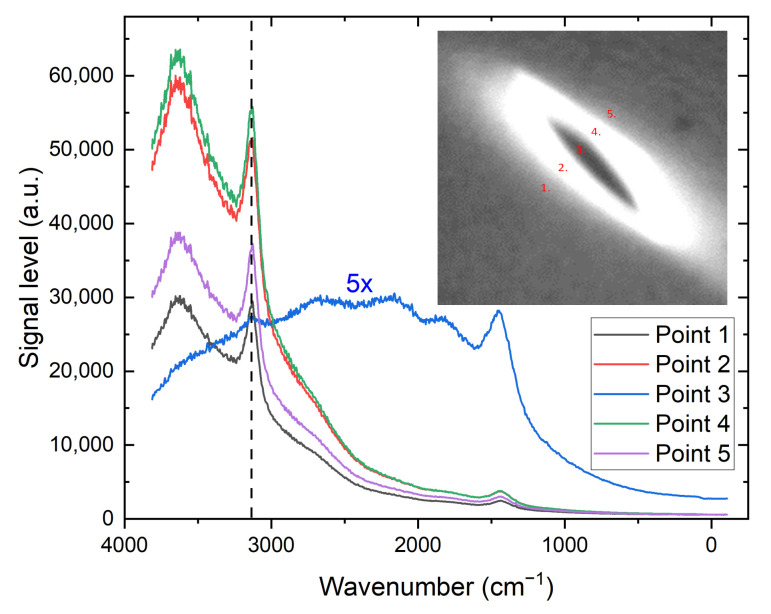
Raman spectra collected in five locations, shown in the inset, at spot 8 of HEN2. The signal for location 3 was magnified five times for clarity. The vertical dashed line represents the peak of the Zero Phonon Line (ZPL) for the nitrogen-vacancy color center (3132 cm${}^{-1}$).

Figure 3 shows the Raman spectra taken for five locations at spot 8. The characteristic Raman mode for a diamond crystals is not visible (1332 cm${}^{-1}$), only the $NV^{0}$ peak is seen at 1428 cm${}^{-1}$ [30]at this particular spot. We attribute it to an overwhelming contribution of the NV signal at this location. The spectra measured outside of the dark area are qualitatively very similar to each other and have a strong peak characteristic to the NV${}^{-}$ color centers at 3132 cm${}^{-1}$. For the midpoint (number 3) the signal was amplified 5x.

## 3. Experiment

**Figure 4:**
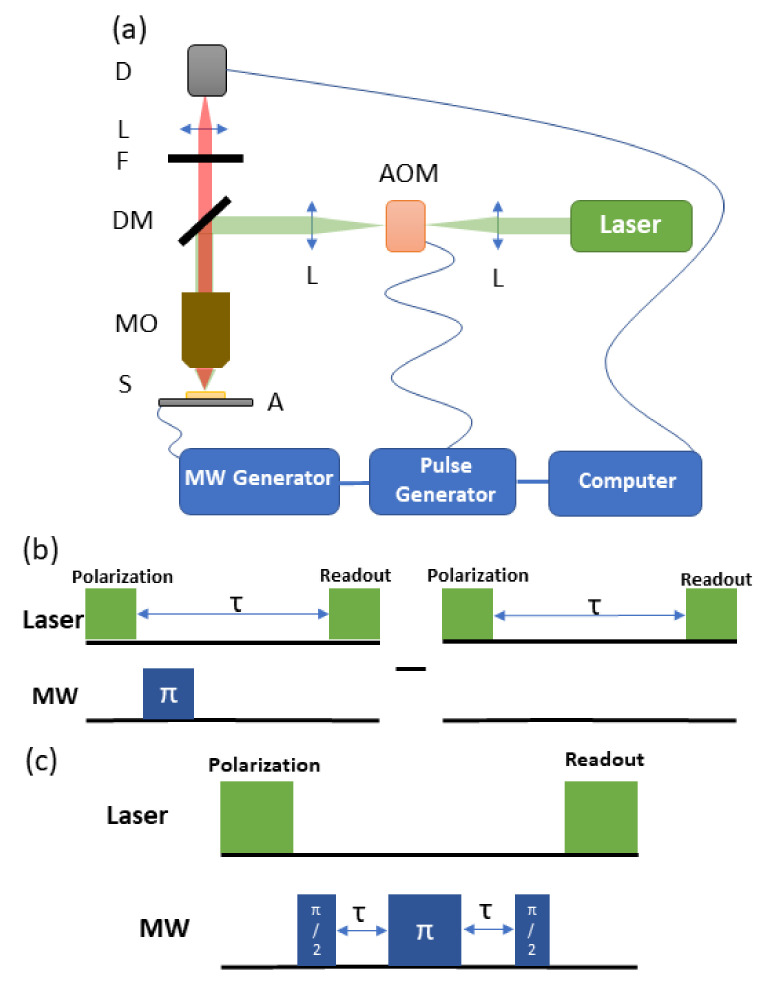
Schematics of the measurement setup: (**a**) the confocal setup, D—detector, L—lens, F—filter, DM—dichroic mirror, MO—microscope objective, S—sample, A—MW antenna, AOM—acusto-optical modulator. The optical and microwave pulse sequence used for $T_{1}$ (**b**) and $T_{2}$ (**c**) measurements. The upper (green) rectangles mark the optical excitation, whereas the lower (blue) ones represent the MW pulses.

The experimental system is a confocal fluorescence microscope shown schematically in Figure 4a. A microscope objective (Olympus Corporation, Shinjuku, Japan, UPLFLN 40×, NA = 0.75, WD = 0.51 mm) was used for focusing the green laser (Lighthouse Photonics, San Jose, Ca, USA, Sprout-G, 532 nm) excitation beam, as well as for the collection of fluorescence light. A dichroic mirror (Thorlabs Inc., Bergkirchen, Germany, DMLP567) and an optical long-pass filter (Thorlabs Inc., Bergkirchen, Germany FEL0600) allow for the detection of light with a wavelength in the range of approximately 600–850 nm using either an avalanche photodiode (Thorlabs Inc., Bergkirchen, Germany, APD130A) or a compact optical spectrometer (Thorlabs Inc., Bergkirchen, Germany, CCS175). Our study was performed in room temperature. Raman spectra were recorded by a spectrometer (Witec GmbH, Ulm, Germany, Alpha 300).

For relaxation time measurements, a microwave (MW) signal of the frequency of 2.87 GHz from a generator (Stanford Research Systems, Sunnyvale, CA, USA, SRS SG396) is fed to a high-power amplifier (Mini-Circuits, Brooklyn, NY, USA, ZHL-16W-43+) and delivered to a loop-gap type antenna structure on a printed circuit board (PCB). The antenna structure is similar to the one described in the reference [31] and is designed to produce a uniform magnetic-field distribution in the range of ∼ 1 mm diameter around its center while maintaining a central hole diameter of 3 mm. Samples were placed on glass coverslips directly on the antenna boards. For the $T_{1}$ and $T_{2}$ measurements, the MW power was delivered in the form of pulses produced and controlled by a programmable pulse generator.

The $T_{1}$ measurements were based on the “relaxation in the dark method”, [12,32], whereas $T_{2}$ was measured with the help of the Hahn spin-echo [33]. Figure 4b,c show the timing sequence of the MW and optical pulses used in the experiments to measure $T_{1}$ and $T_{2}$. For the $T_{1}$ and $T_{2}$ measurements, the length of the microwave π pulse was 50 ns.

## 4. Results

**Figure 5:**
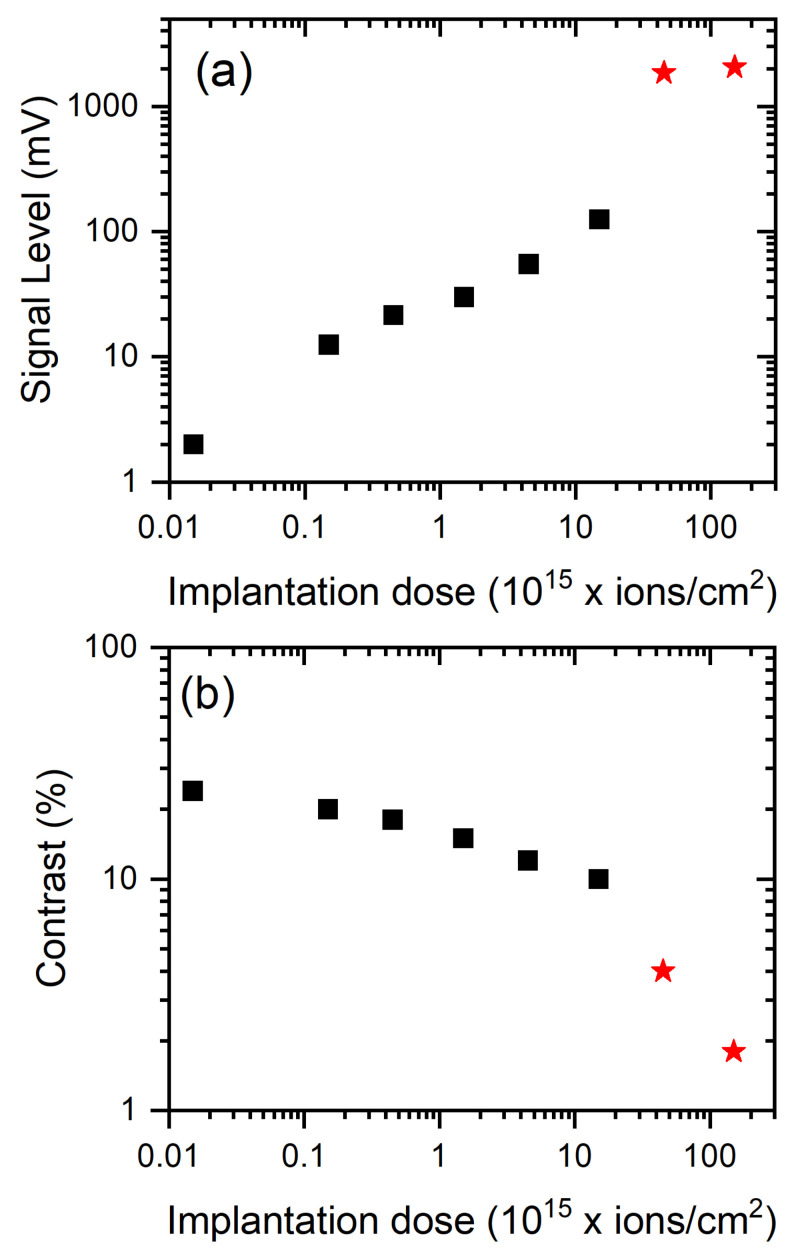
The fluorescence level from different spots versus the implantation dose of protons (black squares-HEN1, red stars-HEN2) (**a**) and the ODMR contrast versus an irradiation dose (**b**).

Our initial measurements focused on the examination of the fluorescence level of samples exposed to various irradiation doses at a constant power of the pumping laser (a 1-mW green laser). As the implantation dose increases, the signal level of fluorescence increases too until no more nitrogen–vacancy color centers can be created. Figure 5a shows this specific case (the black squares for the sample HEN1). These data are consistent with those presented in the paper [25]. The elongated shape of the irradiated areas on HEN2 results from an instability of the proton beam throughout the prolonged implantation. The red asterisks show the results for the sample HEN2. It can be seen that the level of fluorescence for spots 7 and 8 (Figure 1b) is similar to the level of HEN1 though somehow higher than for the trend of HEN1. This is because the implemented doses no longer produce vacancies in the sample but only damage the diamond structure, which may additionally contribute to the background of scattered light. That damage is seen in Figure 1b in the form of a black spot in the middle of the measuring point. A similar observation of a fluorescence–intensity stabilization for high implantation doses was also presented in [34].

In addition to a standard fluorescence measurements, we have also conducted an Optically Detected Magnetic Resonance (ODMR) study with the investigated samples. Figure 5b shows the contrast level of ODMR versus the implantation dose of protons and its decrease with the increase of the dose (the data for points 7 and 8 are lower than the expectation from the HEN1 trend, which is consistent with our interpretation as local sample damage by the stronger doses). A similar effect was observed in Ref. [35], where the influence of local strain in diamond induced by interstitial carbon atoms was studied at a high density of NV centers. The authors of [35] developed a model taking into account the lattice swelling due to interstitial carbon atoms and explained the evolution of ODMR spectra at elevated NV densities. Our findings are also in agreement with this model.

An important consequence of any high-energy particle irradiation is the creation of vacancies in diamond which may later be bound to nitrogen and form the NV centers. After annealing, the NV density is increased, which substantially enlarges the fluorescence intensity observed from the stronger irradiated samples (Figure 1). On the other hand, the local increase in defect density enhances the spin relaxation relative to the non-irradiated area because of the dipole–dipole interactions between NV center and paramagnetic defects that scale with the distance as $r^{-3}$. The NV distance to such defects is, in turn, a function of the density ρ which for low-defect density can be assumed to scale as $r∼\rho^{1/3}$. However, that scaling fails when more impurities occur in the diamond lattice and a number of processes such as annealing, Frenkel-pair recombination, vacancy clustering, migration to the surface, etc. take place. To determine the effect of proton–beam irradiation on relaxation, we measure the longitudinal, $T_{1}$, and transverse, $T_{2}$, relaxation times and their dependencies on implantation doses.

**Figure 6:**
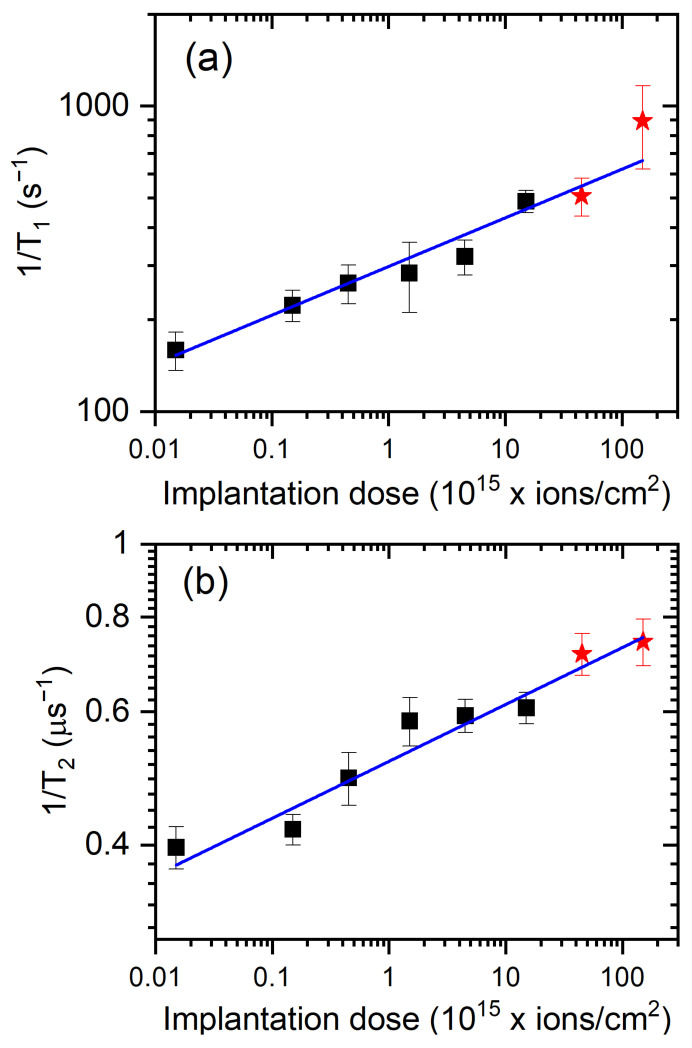
The longitudinal relaxation rate (**a**) and the transverse relaxation rate (**b**) as a function of an implantation dose (black squares-HEN1, red stars-HEN2). Linear functions were fitted to the data on the graphs. The slope for (**a**) was 0.16 and was twice as large as for the data from (**b**) 0.08.

Figure 6 shows the relaxation rates (inverse of the relaxation time) obtained in our experiment. The data for longitudinal relaxation rate [Figure 6a] and the transverse one [Figure 6b] increase with the implantation doses and can be fitted to linear functions (in logarithmic scales). For Figure 6a, the slope of the dose dependence of the $1/T_{1}$ rate was 0.16, which is twice as large as for the $1/T_{2}$ dependence shown in Figure 6b, where the slope was 0.08. These results appear to be consistent with the earlier observations [26,36,37,38,39] where the role of dipolar interaction in dense samples was identified. In Ref. [40], the dephasing solely due to the NV–NV dipolar interaction for [NV] 45 ppm concentration was estimated to be around 2π×100 kHz, which is also consistent with our results after their rescaling to NV density of our samples. When discussing the effects of relaxation, however, the role of other (mainly P1 and N3) centers must not be forgotten at higher densities at higher densities [41,42,43,44].

In Table 2, we collected data for these experiments. The presented references do not contain nitrogen implantation, only the ions used to create the vacancy. The last row in the table is for non-implanted samples.

## 5. Conclusions

In summary, this work presents the characterization of spin relaxation in NV centers produced by 1.8 MeV proton implantation with doses ranging from $1.5\times 10^{13}$ to $1.5\times 10^{17}$ ions/cm${}^{2}$ followed by annealing of the diamond sample. The proton implantation results in an interesting depth profile of the vacancy distribution: starting from 1 micrometer below the surface, the vacancy density slowly increases with the depth up to about 16 µm, where it starts to rapidly increase, peaking at around 20 µm. This opens up a possibility of using proton implantation for creating NV centers with a relatively uniform depth profile when the nitrogen-doped layer is a few micrometers thin. For many sensing applications, thin and dense layers of NV centers are desired in order to maximize the signal while maintaining sufficiently short distance between the source and the sensing spins. Therefore, high-energy protons seem particularly suitable for the creation of vacancies in typically used NV sensing layers that have a thickness of 0.1–10 µm.

Additionally, spectroscopic analysis of the irradiated samples revealed diamond damage caused by the largest applied doses, correlated with the drop of the fluorescence of the affected areas, which indicates limitations of the useful doses.

Finally, the measurements of the longitudinal and transverse relaxation times demonstrated their dependence on the implantation doses, which we attributed to dipole–dipole interactions. These results are comparable with the literature values for similar, nitrogen-rich diamond samples, although implanted with different ions or irradiated with electrons. The presented measurements should help to guide the preparation of microscale diamond NV sensors for magnetometry or bio-magnetometry, either thin diamond plates, or shallow nitrogen-doped layers overgrown on a high quality diamond or in micrometer-sized diamonds.

## Figures and Tables

**Table 1:** Proton–dose values of all implanted spots.

Spot Number	Dose [ions/cm${}^{2}$]
1	$1.5\times 10^{13}$
2	$1.5\times 10^{14}$
3	$4.5\times 10^{14}$
4	$1.5\times 10^{15}$
5	$4.5\times 10^{15}$
6	$1.5\times 10^{16}$
7	$4.5\times 10^{16}$
8	$1.5\times 10^{17}$

**Table 2:** Table comparing type of diamond, initial *N* concentration, energy of incident particles, type of implantation, depth of penetration, dose, value of longitudinal, and transverse relaxation time for selected references.

Type of Diamond	Initial *N* Concentration [ppm]	Energy [MeV]	Type Implantation	Depth [μm]	Dose [cm${}^{2}$]	$T_{1}$ [ms]	$T_{2}$ [μs]	Authors
HPHT	200	0.2	electron	20	$1.1\times 10^{19}$–$2.5\times 10^{21}$	4.8–0.83		Jarmola et al. [36]
CVD	0.1	1	electron		$1\times 10^{15}$–$5\times 10^{19}$	5.5	100–300	Alsid et al. [41]
CVD	10	1.8	He${}^{+}$	1	$1\times 10^{15}$		1 ($T_{2}^{*}$)	Wojciechowski et al. [45]
HPHT CVD	50–0.5	0.05	He${}^{+}$	0.25	$1\times 10^{10}$		0.3 ($T_{2}^{*}$)	Trofimov et al. [37]
CVD	0.001	0.15–0.05	He${}^{+}$	0.1	$1\times 10^{9}$–$1\times 10^{13}$		1.5 ($T_{2}^{*}$)	Kleinsasser et al. [46]
CVD	0.005	2	He${}^{+}$	3.4	$1\times 10^{12}$–$1\times 10^{15}$		150	Genish et al. [47]
CVD	200	2	proton	25	$1\times 10^{15}$–$1\times 10^{16}$			Jin et al. [26]
HPHT	50	1.8	proton	20	$1.5\times 10^{13}$–$1.5\times 10^{17}$	6–1.25	2.5–1.1	This paper
HPHT CVD	100 0.001		non implantation		$1\times 10^{10}$		1 300	Rodin et al. [48]

## Data Availability

The data presented in this study are available on request from the corresponding author.
